# Think twice before stent insertion for renal artery aneurysm with elusive etiology: a case report

**DOI:** 10.1186/s12893-019-0622-5

**Published:** 2019-10-26

**Authors:** Jian-zhong Zhang, Peng Zhang, Li-yang Wu, Yong Wang, Kun Gao, Qiang Huang, Xiao-hui Wang

**Affiliations:** 1 Department of Urinary Surgery, Beijing Chaoyang Hospital, Capital Medical University, Beijing, China; 2 Department of Interventional Radiology, Beijing Chaoyang Hospital, Capital Medical University, Beijing, China; 3 Department of Pathology, the 1st Affiliated Hospital of Zhengzhou University, Zhengzhou, Henan China

**Keywords:** Renal artery aneurysm (RAA), Stent insertion, Embolization, Undifferentiated sarcoma

## Abstract

**Background:**

Endovascular treatment has been recognized as the first line therapy for renal artery aneurysm (RAA). However, RAA related with malignancies had been sporadically reported in the literature. Stent insertion should be contraindicated for RAAs with malignant etiology, whereas surgery be optimal.

**Case presentation:**

A 40-year-old female underwent covered stent insertion to exclude the left RAA for suspected Takayasu arteritis in a reginal hospital. Three months later the RAA recurred with sign of threatened rupture, and the patient was transferred for salvage embolization with coils and thrombin injection. However, 20 days after the embolization procedure, multiple painful subcutaneous nodules developed in her flanks. Undifferentiated sarcoma was revealed by the pathological biopsy of the nodules. The RAA in this case was most likely related with the malignancy.

**Conclusion:**

Malignancy was the most likely etiology behind recurrent aneurysm in this case. Definite diagnosis is mandatory for interventional radiologists before stent insertion for treatment of RAA.

## Background

Renal artery aneurysm (RAA) is one of the most common visceral artery aneurysms, requiring treatment because of the high risk of rupture [1]. Surgery repair of RAA is often complex and technically challenging, with some may even require nephrectomy. Endovascular treatment has been recognized as the first line therapy for RAA [2, 3]. However, RAA related with malignancies had been sporadically reported in the literature [4, 5]. Stent insertion should be contraindicated for RAAs with malignant etiology, whereas surgery be optimal. Here we present a case of recurrent RAA after prior covered stent insertion, with elusive etiology. Salvage embolization had to be performed with the sacrifice of the left kidney. Multiple subcutaneous nodules developed, and pathological biopsy revealed undifferentiated sarcoma.

## Case presentation

**Fig. 1:**
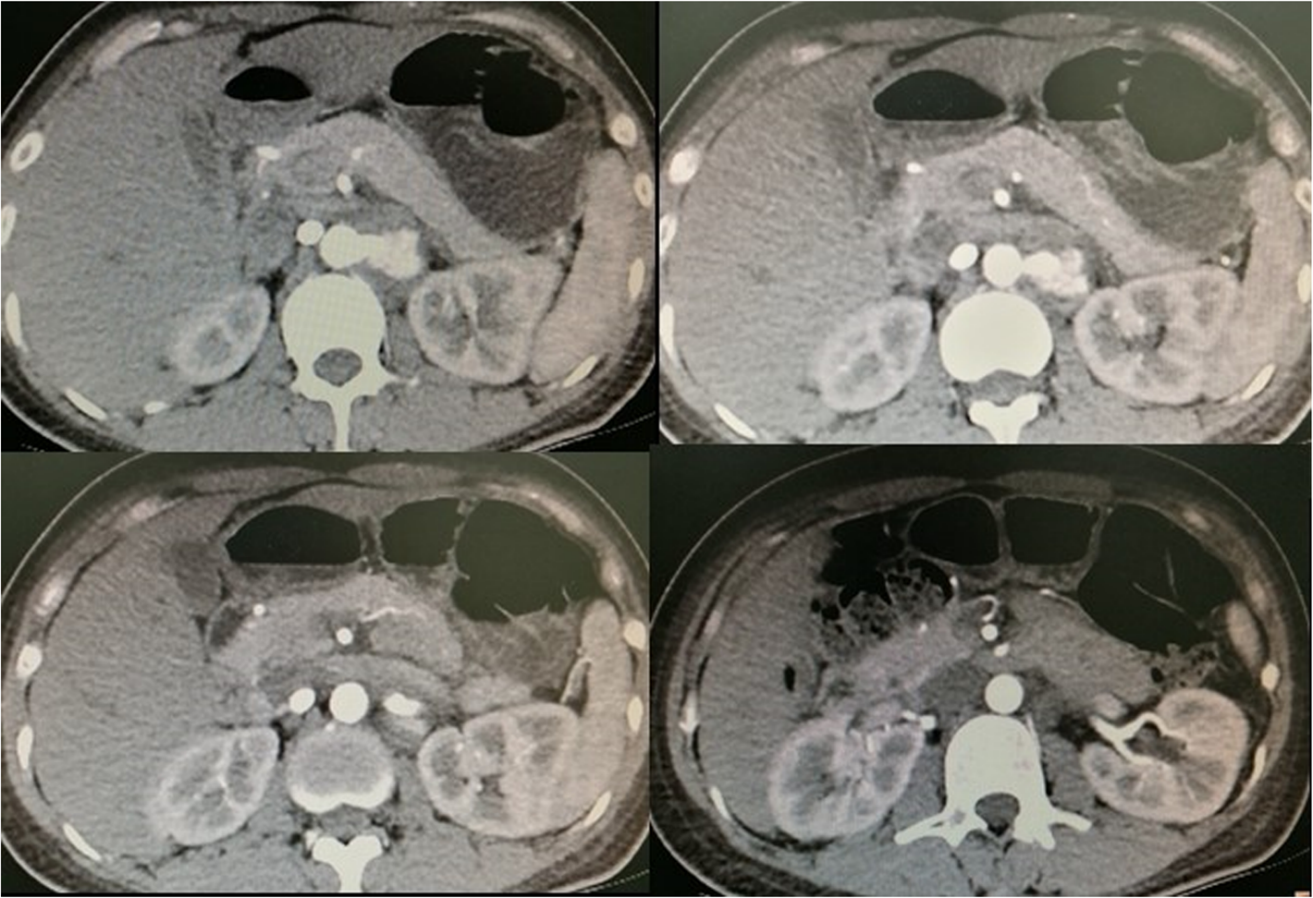
Computed tomography (CT) revealed aneurysm in the left renal artery

**Fig. 2:**
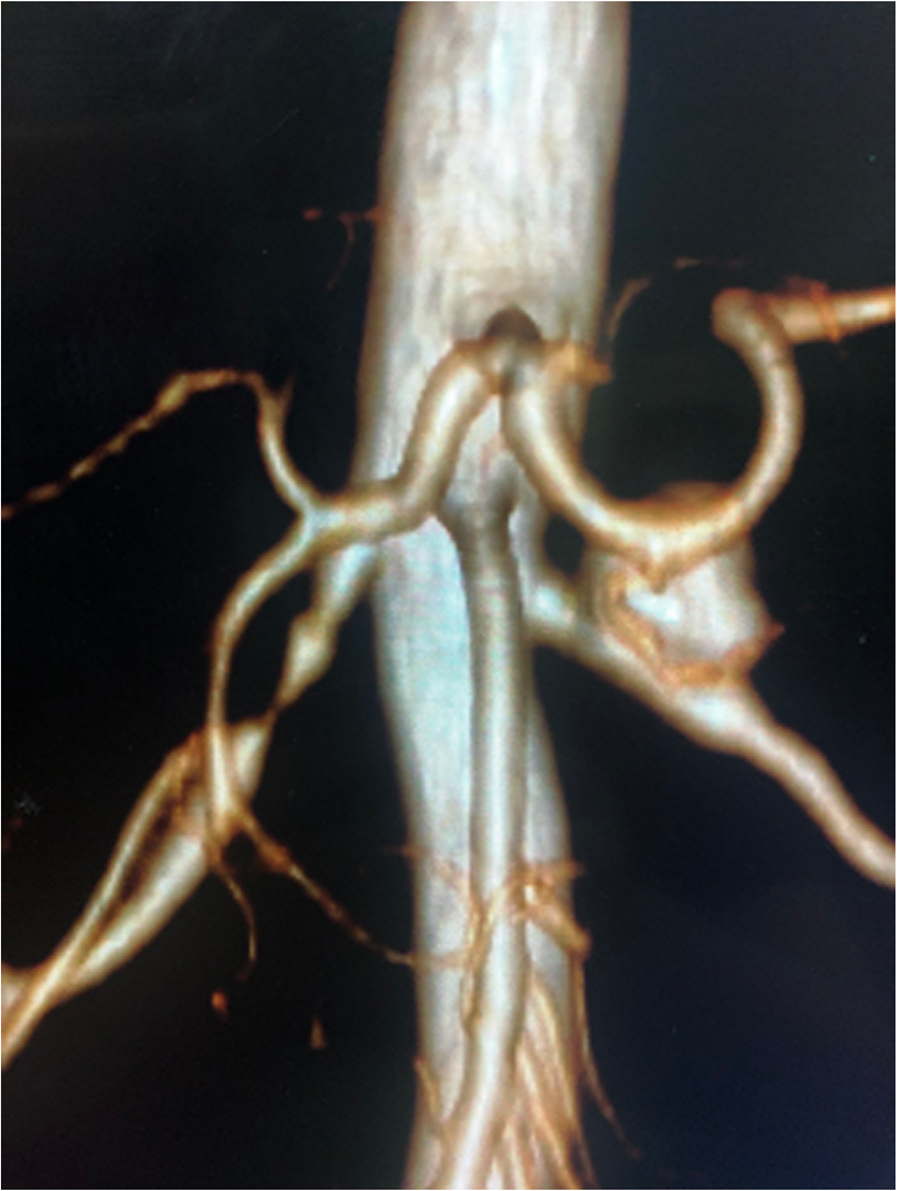
Pre-procedural Reconstructed CT showed the stenosis in the right renal artery and aneurysm in the left

**Fig. 3:**
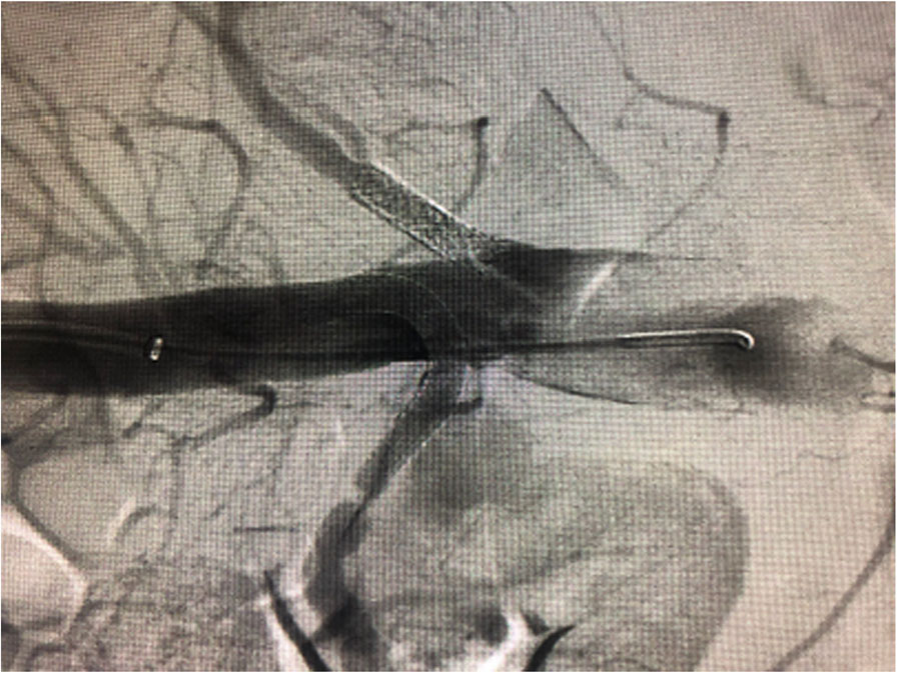
Complete angiography during the first interventional procedure showed the bilateral stents. A stenosis was noted distal to the covered stent in the left renal artery

**Fig. 4:**
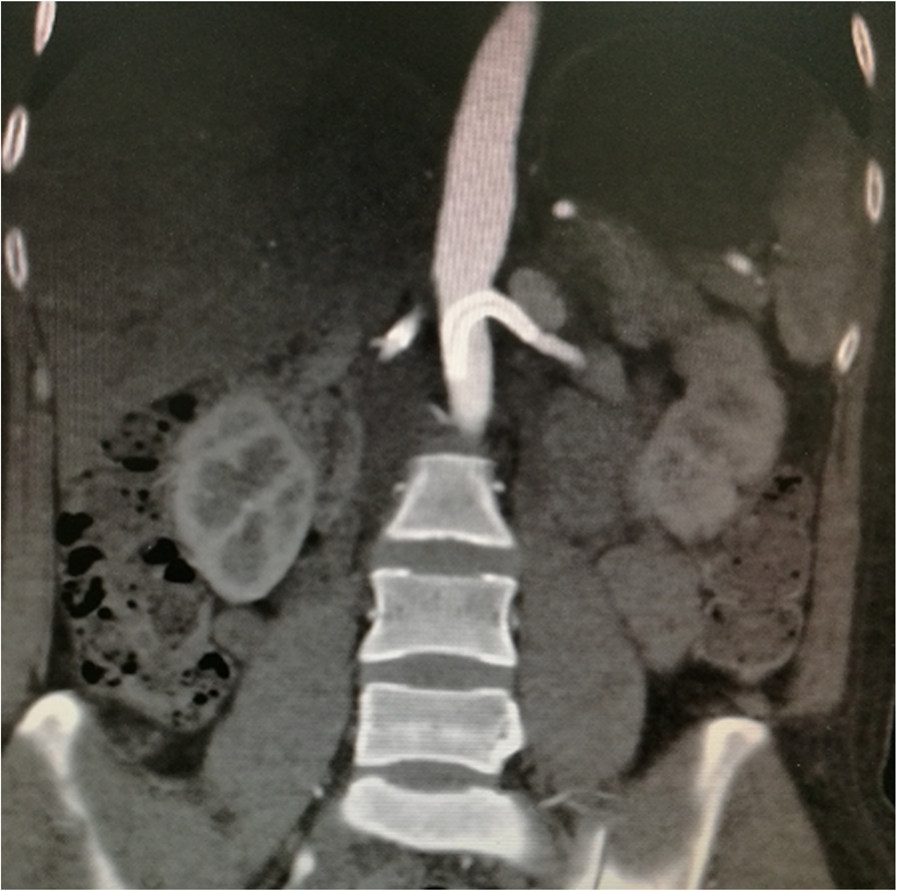
Reconstructed CT revealed the left covered renal artery stent stretching into the aorta

**Fig. 5:**
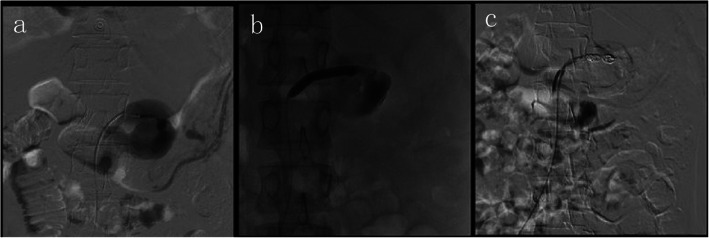
Angiography showed the bilateral renal artery stent insertion, and the presence of a large aneurysm originating from the distal end of the left renal artery stent (**a**). A 6 × 40 mm balloon catheter was placed into the stent, and thrombin injection was completed under protection of the balloon dilation (**b**). Further embolization with 2 coils placed into the stent (10 mm, Boston Scientific, Interlock) successfully excluded the aneurysm, confirmed by the angiography check of the left renal artery (**c**)

A 40-year-old female was initially admitted into a regional hospital for the left flank pain and refractory hypertension. Ultrasound and computed tomography (CT) revealed severe stenosis in the right renal artery and aneurysm in the left (Figs. 1 and 2). The diagnosis of Takayasu arteritis was assumed, but without evidence from either lab tests or images. Endovascular bilateral renal artery stenting was performed, with uncovered stent inserted in the right renal artery and covered one in the left (Figs. 3 and 4). A stenosis distal to the covered stent in the left renal artery was noted and could be the potential cause of recurrence (Fig. 3). The symptoms ameliorated after the interventional therapy. However, three months later the symptom of left flank pain recurred, and repeated CT showed relapse of the left RAA. Expectant treatment was ineffective, so the patient was transferred to our Urology Surgery Department. The patient complained about fatigue and intermittent dull pain in her left flank. But general condition was moderate. Multidisciplinary consultation was organized following the admission of the patient in our center. The size of the aneurysm (over 30 mm in diameter) and progressive pain gave the impression of a threatened rupture. Given the giant size of the aneurysm, previous covered stent implantation history, and the elusive etiology, immediate radical nephrectomy was considered, however, the left covered renal artery stent stretched into the aorta was a great challenge for surgery (Figs. 3 and 4). She was transferred to the interventional radiology suite for endovascular treatment. Written informed consent was obtained. Under local anesthesia, access from the right groin was obtained and a 5F sheath was placed. Angiography showed the bilateral renal artery stent insertion, and the presence of a large aneurysm originating from the distal end of the left renal artery stent (Fig. 5a). The stent was a covered one and the proximal part of it stretched into the aorta. A 6 × 40 mm balloon catheter (ATB advance, Cook, Bloomington, IN, USA) was placed into the stent through a guide wire (Terumo, Tokyo, japan). Thrombin injection was completed under protection of the balloon dilation (Fig. 5b). Further embolization with 2 coils placed into the prior stent (10 mm, Boston Scientific, Interlock) successfully occluded the aneurysm, with sacrifice of the left kidney (Fig. 5c).

**Fig. 6:**
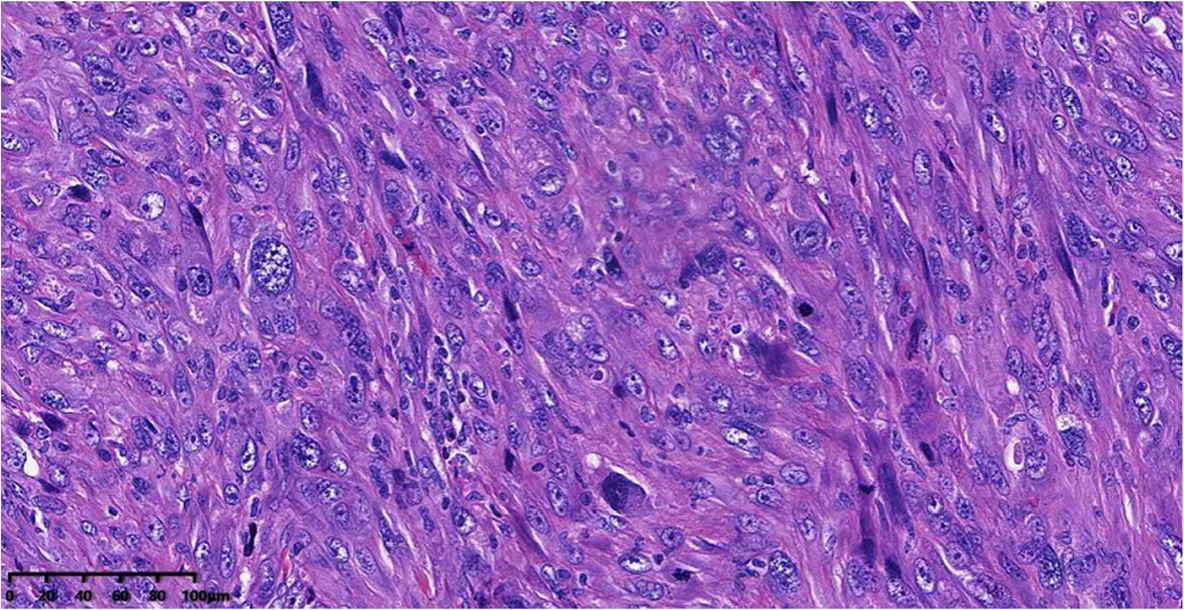
Microscopic examination of the tumor. Spindle shaped tumor cells with marked pleomorphism and atypia (H&E × 20), indicating undifferentiated sarcoma

The one-week post-embolization renal function tests (BUN and CREA) were within normal range. The patient remained stable after the second endovascular treatment and was discharged. However, the patient developed multiple painful subcutaneous nodules in her flanks 20 days after the embolization procedure. Biopsy was performed in the regional hospital and pathological exam revealed undifferentiated sarcoma (Fig. 6). Chemotherapy was tried but the patient could not tolerate it. The patient experienced more episodes of hemorrhagic shock which required multiple blood transfusions, and finally passed away 2 more weeks later.

## Discussion and conclusion

RAA rupture is a critical clinical situation and requires emergency management. Surgery is limited for the deep vascular anatomy and poor vision. Varied endovascular approaches including embolization coils, covered stent grafts, flow-diverting stents, vascular plugs, thrombin or liquid embolic agents have been reported [2]. A covered stent may be the appropriate and reliable choice for the treatment of a ruptured RAA, with advantage of kidney preservation. However, stent implantation was not a good choice for RAA with malignant etiology. Surgery was contraindicated due to the prior covered stent when the RAA recurred in this case, the salvage embolization only delayed the rupture of the aneurysm. Multiple blood transfusions during the follow up indicated further hemorrhage from the aneurysm rupture occurred. We had decided to embolize the left renal artery only after a detailed discussion with the urologists in our center. Nephrectomy was proposed, but the risk of failing to ligate the left renal artery or relapse of aneurysm in the proximal part of the ligation was great, because of the prior inserted stent. Hemorrhage and exudation from the aneurysm could pose technical difficulty to expose renal hilar. Therefore, embolization with coils and thrombin injection surely was not the optimal choice, but the safest and simplest one in the situation.

For this case, the oversizing of the covered stent (Fig. 3) might be one of the culprits for recurrence of the aneurysm. The length of the stent was also not well considered, which led to great challenge for both surgery and repeated interventional procedure. But in our opinion, even with suitable stent choice, the malignant nature of the renal artery lesion must lead to recurrence sooner or later.

RAA caused by malignancies had been reported in the literature. A giant RAA caused by malignant solitary fibrous tumor of the renal vein was described by Hertz AM et al. [6]. RAA caused by sarcoma had also been reported [4, 5]. The possibility that the coincidental association between the aneurysm and undifferentiated sarcoma could not be totally excluded, which was a great limitation for this case.

Sarcoma of arteries are rare and most frequently seen in elastic arteries, they are typically intraluminal, and have a variety of clinical presentations, while are rarely suspected clinically [4]. The tumor had aggressive behavior, showing evidence of distant metastasis [4]. All the above characteristics were consistent with our case. Unfortunately, no direct biopsy or pathological exam from the renal artery was available, but malignancy was still the most likely etiology behind recurrent RAA in this case. The prognosis of this case would be greatly improved if the operators had thought twice or proposed surgery before the stent insertion, since there was no solid evidence of Takayasu arteritis. After all, definite diagnosis is mandatory for interventional radiologists before stent insertion for treatment of RAA. This case highlights the potential that malignancy should be excluded in the diagnosis of aneurysm without obvious evidence supporting benign etiologies.

## Data Availability

Not applicable.

## Abbreviations

CT: Computed tomography
RAA: Renal artery aneurysm
